# The Potential Role of miRNAs as New Biomarkers for Osteoporosis

**DOI:** 10.1155/2018/2342860

**Published:** 2018-05-06

**Authors:** Maria Materozzi, Daniela Merlotti, Luigi Gennari, Simone Bianciardi

**Affiliations:** ^1^Department of Medicine, Surgery and Neurosciences, University of Siena, Siena, Italy; ^2^Department of Medical Biotechnologies, University of Siena, Siena, Italy; ^3^Division of Genetics and Cell Biology, Age Related Diseases, San Raffaele Scientific Institute, Milan, Italy; ^4^Fondazione Umberto di Mario ONLUS, Toscana Life Sciences, Siena, Italy

## Abstract

Osteoporosis is the most common metabolic bone disorder affecting up to 40% of postmenopausal women, characterized by a reduction in bone mass and strength leading to bone fragility and fractures. Despite the available tools for diagnosis and stratification of a fracture risk, bone loss occurs insidiously and osteoporosis is often diagnosed after the first fracture has occurred, with important health-related outcomes. Therefore, the need of markers that could efficiently diagnose bone fragility and osteoporosis is still necessary. Over the past few years, novel studies have focused on miRNAs, small noncoding RNAs that are differentially expressed in many pathological conditions, making them attractive biomarkers. To date, the role of miRNAs in bone disorders remains in great part unclear. In particular, limited and partly conflicting information is available concerning their use as potential biomarkers for osteoporosis, due to differences in patient selection, type of samples, and analytical methods. Despite these limits, concordant information about some specific miRNAs is now arising, making likely their use as additional tools to stratify the risk of osteoporosis and possibly fractures. In this review, we summarize the most relevant studies concerning circulating miRNAs differentially expressed in osteoporotic patients along with their function in bone cells and bone turnover.

## 1. Introduction

Osteoporosis is the most common metabolic bone disorder in humans and is characterized by a decrease in bone mass and quality leading to decreased bone strength [1]. It is estimated that up to 40% of postmenopausal women and 20% of men over 50 years may be affected worldwide, with millions of fractures registered every year [2]. Considering the aging population, these numbers are expected to increase steadily over the next years, making osteoporosis a major health-economical issue worldwide [3].

Bone is a metabolically active tissue in which the process of remodeling is continuously carried on throughout life. Under normal conditions, osteoblasts (the bone-forming cells) and osteoclasts (the bone-resorbing cells) operate in a well-organized and strictly controlled manner, thus ensuring the renewal of bone tissue in a normal skeletal structure [4, 5]. Remodeling also protects bones from the occurrence of damage by adapting their structure and strength to the circumstantial loading requirements [4]. Aging, as well as the presence of predisposing conditions, may cause either an unbalance in bone remodeling with an increased bone resorption not equally compensated by bone formation, or an increased remodeling velocity, leading to low bone mass, reduced strength, and deterioration of the skeletal microarchitecture [6]. These pathological aspects lead to bone fragility and consequent increased risk of fractures, most commonly involving the forearm, vertebral bodies, and hip [7].

Since many years, the most used method for the diagnosis of osteoporosis and the prediction of a fracture risk consists in the measurement of bone mineral density (BMD), assessed by dual energy X-ray absorptiometry. In fact, the risk of fragility fractures increases progressively as BMD declines [8]. However, several other components of bone strength affecting either the structural or material properties of bone have been identified that are not necessarily captured with the measurement of BMD. Moreover, the use of markers of bone formation such as serum procollagen type I N-terminal propeptide (s-PINP) and bone resorption such as serum C-terminal telopeptide type I collagen (s-CTX) and urinary N-telopeptide (NTX) is common in the clinical practice [9]. These markers have been developed to provide a noninvasive assessment of bone turnover in different skeletal pathologies and have helped the clinicians to identify patients at a high risk for fractures and to monitor the efficacy of therapies [10]. Nevertheless, to date, s-CTX and s-P1NP show specific limitation such as lack of normative reference population databases, inadequate standardization of quality control, sample handling, and poor association with bone strength and fracture risk [11]. Despite the innovations introduced in the field of diagnostics, bone loss occurs insidiously and it is initially asymptomatic, so that osteoporosis is often diagnosed after the first clinical fracture, with consequent reduction of autonomy and increased mortality [12, 13]. Moreover, these patients often require hospitalization that increases the onset of several complications. In this context, the study of new potential biomarkers which can be used alone or in combination with existing markers, allowing an early and efficient diagnosis before the occurrence of fractures and an evaluation of the patient's response to therapy, would prove to be of great interest, for both clinical practice and translational research [11, 14].

Osteoporosis is a complex and multifactorial disorder with a recognized hereditary component, although genetic variants associated with the disease have limited impact on gene expression and explain only a small fraction of the disease etiology [15]. Thus, the study of new epigenetic factors, connected with this pathology, may increase our knowledge about its pathogenesis and epidemiology.

## 2. Epigenetics of Osteoporosis

Epigenetic mechanisms include DNA methylation and histone modifications that regulate gene transcription and noncoding RNA (ncRNA) that act at a posttranscriptional level. In fact, while 70–90% of human genome is transcribed into RNA, only 1-2% of these RNAs encode for proteins, suggesting that ncRNA represents most of human transcriptome [16, 17].

Among known epigenetic mechanisms, microRNAs (miRNAs) are one of the most studied regulators of gene expression in both physiological and pathological conditions [14]. miRNAs are noncoding, single-stranded RNA of about 22–24 nucleotides found in both plants and animals and act at a posttranscriptional level, directly modulating their target mRNA through the formation of an RNA-induced silencing complex [18]. They negatively regulate their targets in two ways, depending on the degree of complementarity between the miRNA and the target sequences within 3′ untranslated regions (UTRs) of mRNA. miRNAs that bind with perfect complementarity with the targeted mRNA sequence induce the RNA interference pathway that leads to the degradation of mRNA [19–22]. miRNAs that bind their almost complementary target sequences within 3′ UTRs of mRNA repress gene expression posttranscriptionally at the level of translation, without degradation of mRNA [23, 24].

In the past decade, a lot of evidence showed that also long noncoding RNAs (lncRNAs), ncRNA longer than 200 nucleotides, play important roles in diverse biological process, such as cell growth, transcriptional regulation, tumorigenesis, and stem cell development [25, 26]. Despite the increasing amount of data about the role of lncRNA in the epigenetic regulation of transcription, their role in skeletal basic and clinical biology remains largely unknown [27]. Unfortunately to date, only few studies have investigated the role of some lncRNAs (ANCR, H19, MEG, DANCR, etc.) on bone metabolism and they principally focused on osteoblast differentiation and function, without taking in consideration the role of these ncRNAs as biomarkers for osteoporosis [28–30].

The key role of miRNAs in the regulation of bone homeostasis and metabolism is well established in a lot of experimental observations, showing how these noncoding RNAs may affect osteoblast or osteoclast differentiation, function, apoptosis, and proliferation. This role was first demonstrated *in vivo* using mice knockout for Dicer (protein necessary for the maturation of miRNA) in chondrocytes; these mice showed a reduction in skeletal size and died at weaning [31]. In support of this finding, other two observations demonstrated the fundamental role of miRNAs during normal skeletal development. In the first of these studies, the deletion of Dicer in osteoprogenitor cells impaired bone formation and caused embryonic lethality, while the other study showed that the ablation of Dicer in mature osteoblasts leads to an increased bone mass phenotype [32]. Finally, when we look for new biomarker, it is important to consider that the choice between serum and plasma as starting material and the quality control determination are a crucial point for the success of the experiments [33]. To date, many papers have been published in order to answer the questions on what are the best laboratory practices, the best experimental conditions, and the best starting samples to obtain good biomarkers including miRNAs. In one of these papers, Blondal and colleagues identified 119 miRNAs that are most commonly present in human serum and plasma samples, and for each of them, they developed a normal reference range [34]. Interestingly, some of these miRNAs were found differentially expressed in osteoporotic patients. However, some other miRNAs identified as potential markers of osteoporosis appear to be less common in normal conditions, and, perhaps, they could represent a more specific signature of the disease. A list of such miRNAs is shown in Table 1. Thus, given the great importance of miRNAs as regulators of bone homeostasis and remodeling in both physiological and pathological conditions, the purpose of our review is to summarize what we known so far regarding circulating miRNA in patients with osteoporosis.

## 3. Diagnostic Biomarkers of Osteoporosis

Accumulated evidence indicated that miRNAs in many cases define the physiology nature of the cell and play significant roles in the regulation of diverse biological processes, such as development, cell differentiation, proliferation and death, immunity, and metabolism [35, 36]. Furthermore, aberrant miRNA expression should proportionally affect those critical processes and has been implicated in a wide variety of human diseases including cancer, viral infections, nervous system disorders, cardiovascular and muscular disorders, and diabetes [36, 37]. This implies that the use of these aberrantly expressed miRNAs as biomarkers for diseases is not only a valuable diagnostic strategy but it also makes these ncRNAs good candidates for new drug discovery [35, 36].

Furthermore, the presence of several miRNA quantification platforms and the introduction of high-throughput technologies, such as miRNA microarray, Real-Time PCR TaqMan Array microfluid cards, locked nucleic acid- (LNA-)-based high-throughput PCR, and next generation sequencing (NGS), facilitated the analysis of circulating miRNA expression profiles [38]. To date, these methodologies have replaced low-throughput analysis (Northen blotting and cloning) that present a lot of limitation due to their poor quantification output, time-consuming activity, and relatively low sensitivity and are widely used in initial screening of circulating miRNA and for generation of signatures from body fluid in numerous diseases [38, 39].

In this context, the possibility of detecting miRNAs as diagnostic markers of osteoporosis is certainly appealing for the clinical practice, and over the years, it has led to a rapid increase of studies that not only aimed at understanding the function of miRNAs in bone cells, but also understanding their potential as circulating biomarkers and identifying interesting candidates.

**Figure 1:**
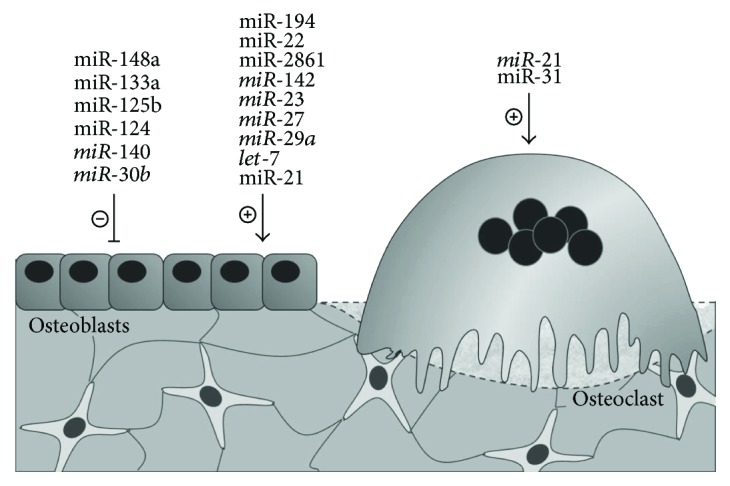
Circulating miRNAs differentially expressed in osteoporotic patients, either upregulated (roman) or downregulated (*italic*), and their functions studied in bone cells.

Although some of these findings are promising, a unifying method of analysis is missing, outlining a complicated and chaotic picture. Important discrepancies are found between studies, regarding the type of samples (e.g., plasma, serum, or whole blood) or populations used as control groups (e.g., healthy, osteopenic, and osteoarthritic subjects). The analysis is also carried out in different ways considering either a different number of miRNAs evaluated or platforms used, as well as reference genes used to normalize the analysis. Despite these limits, different studies showed partly concordant results, identifying miRNAs that appear to be differentially expressed in osteoporosis, with specific targets and functions on bone turnover, as demonstrated by experimental analyses. Here, we present the most relevant findings regarding circulating miRNAs as diagnostic biomarkers in osteoporosis, trying to describe, when possible, their functions and targets identified at the bone level. A complete list of these miRNAs, along with their biological functions and potential targets, is summarized in Tables 2 and 3 and Figure 1.

### 3.1. miR-21 and miR-148a

In several studies, a differential expression of miRNAs in the serum of patients can indeed effectively discriminate osteoporotic and nonosteoporotic patients. For example, Seeliger et al. discovered 9 upregulated circulating miRNAs (miR-21, miR-23a, miR-24, miR-93, miR-100, miR-122a, miR-124a, miR-125b, and miR-148a) that could significantly distinguish between serum samples of osteoporotic and nonosteoporotic fractured patients in a cohort of 30 subjects per group [40]. At a cellular level, two of these miRNAs, in particular, miR-21 and miR-148a, are known to play specific roles in bone homeostasis.

miR-21 affects both osteoclasts and osteoblasts. This miRNA is highly expressed during osteoclastogenesis and promotes the differentiation of murine BMMs through the downregulation of PDCD4 (a repressor of OC differentiation) and the survival of mature osteoclasts by the downregulation of FasL (involved in the Fas/FasL pro-apoptotic pathway [41]. It is likely that, at least in part, through this mechanism, estrogen signaling inhibits miR-21 biogenesis and promotes osteoclast apoptosis. This finding further highlights the relevant role of miR-21 in these cells [42]. In osteoblasts as well, miR-21 promotes differentiation and mineralization in MC3T3-E1 cells, targeting the expression of Smad7, a repressor of proliferation, differentiation, and mineralization of osteoblasts [43, 44].

Similarly, miR-148a promotes osteoclast differentiation by directly targeting MAFB, a RANKL-inhibiting protein [45]. In a recent study, it was also shown to inhibit ST2 cell differentiation toward the osteogenic linage by directly targeting lysine-specific demethylase 6b (Kdm6b), a regulator of osteoblast differentiation [46]. Both these miRNAs were also confirmed to be significantly deregulated in the plasma of osteoporotic women in different subsequent studies [47, 48]. However, miR-21 showed opposite modulation and miR-148a is also considered a strong diagnostic marker of osteosarcoma, thus questioning the potential specificity of such miRNAs for osteoporosis [47, 49].

### 3.2. miR-31 and miR-194

Other miRNAs are also potential candidates (miR-130b-3p, miR-151a-3p, miR-151b, miR-194-5p, miR-590-5p, and miR-660-5p) and display higher levels in the blood of osteoporotic women compared to osteopenic women, and, interestingly, expression levels of miR-151b and miR-194-5p were also negatively correlated with femoral neck T-scores [50]. Another miRNA, miR-31, also stands out for being upregulated in both osteoporotic women and men compared to controls. miR-31 has in fact shown higher levels in the plasma of both osteoporotic women (with a fold change increasing with age) and osteoporotic men [51].

While the role of miR-151b on bone homeostasis remains yet unknown, both miR-194 and miR-31 have been shown to play a role in bone biology. miR-194 promotes osteoblast differentiation and activity in studies performed on mouse bone mesenchymal stem cell (BMSCs) cultures, by regulating Runx2 nuclear translocation through STAT1 inhibition and by downregulating COUP-TFII mRNA levels, therefore driving mesenchymal cell differentiation towards osteoblasts instead of adipocytes [52, 53]. miR-31 is highly expressed during RANKL-induced osteoclastogenesis, and its inhibition in murine bone marrow-derived macrophages impairs terminal differentiation and bone resorption activity by targeting RhoA, a GTPase that plays a key role in a crucial step of OC formation, namely the acting ring assembly [54].

### 3.3. miR-133a and miR-27

In a large study in 120 Chinese postmenopausal women who were divided into three groups (normal, osteopenia, and osteoporosis) according to BMD measurement, miR-133a was found upregulated in the plasma of osteoporotic versus osteopenic patients and negatively correlated with hip and spine BMD [47].

In a more recent analysis, 33 miRNAs were found to be downregulated in the serum of osteoporotic women compared to controls (*n* = 5 each), of which miR-27 showed the strongest reduction, as further validated in a large cohort of 81 women with postmenopausal osteoporosis. However, the controls were younger and thus not age-matched [55].

Indeed, both miRNAs have been also reported to have specific and contrasting functions in osteoblast cell lines. miR-27 directly targets adenomatous polyposis coli (APC) gene expression in hFOB1.19 cells (human fetal osteoblastic cell line), leading to *β*-catenin accumulation and consequently activating Wnt signaling, the most relevant pathway for osteoblast formation [56]. Conversely, miR-133 is involved in inhibiting osteoblast differentiation, through the downregulation of Runx2 expression, a downstream effector of Wnt signaling pathway [57].

### 3.4. miR-30b and miR-142

In a more complex analysis involving either animal models of osteoporosis (rats and rhesus monkeys) and postmenopausal women, miR-30b-5p was significantly downregulated in ovariectomized rats and bed-rest rhesus monkeys as well as in the serum of women with low bone mass, together with miR-142-3p and miR-103-3p. Moreover, all these miRNAs positively correlated with the BMD of these patients, making them potential attractive noninvasive biomarkers for osteoporosis [58]. Of interest, miR-30 family miRNAs, including miR-30b, are known *in vitro* negative regulators of BMP-2-induced osteogenesis, inhibiting osteoblast differentiation through targeting of Smad1 and Runx2 expression [59]. Instead, miR-142-3p positively regulates osteoblast differentiation and promotes Wnt signaling by inhibiting APC, similarly to miR-27, as described above [60].

## 4. miRNAs and Osteoporotic Fractures

By a different approach, Bedene et al. identified a correlation between plasma miR-423-5p levels not only with low BMD values but also with the 10-year probability of major fracture in postmenopausal women, as assessed by the fracture risk assessment tool (FRAX) [48].

Moreover, specific circulating miRNAs have been also correlated with the onset of fractures, the most relevant consequence of osteoporosis, in different studies [2]. Such markers could thus provide some insights on fracture risk, with the potential to specifically identify patients with a history of osteoporotic fractures. In this context, specific miRNA profiles have been found in women with osteoporotic fractures, although, some of these studies were performed comparing osteoporotic women with fractures to healthy controls, or to osteoarthritic women. Thus, the miRNAs identified in such comparisons may actually reflect differences between osteoporotic and nonosteoporotic patients including subjects with osteoarthritis, rather than the actual risk of fracture in osteoporotic patients.

Some of the miRNAs we discuss in this review have been also found to be differentially modulated within the bone tissue of osteoporotic patients, and, although they cannot be considered circulating biomarkers, this aspect further supports their diagnostic value. For example, RNA was extracted from the bone tissue of fractured osteoporotic and nonosteoporotic bone, leading to the identification of 5 miRNAs upregulated in the osteoporotic bone, including miR-21, miR-23a, miR-24, miR-100, and miR-125b, with respect to nonosteoporotic bone [40]. These miRNAs were also differentially expressed in the serum, as previously mentioned, suggesting that the deregulation found in the serum reflects and associates with a similar deregulation within the bone tissue.

### 4.1. Single miRNAs Modulated in Fractured Osteoporotic Patients

In a small scale study, specifically designed to compare serum miRNA expression profiles between osteoporotic patients with fractures and osteoarthritic controls, 3 miRNAs (miR-122-5p, miR-125b-5p, and miR-21-5p) were positively correlated with fracture prevalence [61]. In the same study, circulating miR-21-5p levels were also positively correlated to those of CTx, a marker of bone resorption. Conversely, in a different study, miR-21-5p levels appeared significantly reduced among osteoporotic/osteopenic women with vertebral fractures (66% sensitivity, 77% specificity in distinguishing women with a vertebral fracture) than in nonfractured controls [62]. While the exact function of both miR-423 and miR-122-5p in bone cells remains unknown, miR-21, as described before, can influence both osteoclast and osteoblast differentiation processes, whereas miR-125b was shown to play a role in osteoblastogenesis, by inhibiting cell proliferation of ST2 cells (murine mesenchymal cells) induced with BMP-4 [63].

A more recent in-depth analysis studied circulating miRNAs in the sera of patients with idiopathic osteoporosis and fractures versus healthy controls. This analysis uncovered 19 miRNAs significantly regulated in premenopausal or postmenopausal women and male idiopathic osteoporosis, compared to age-matched healthy individuals. Eight of these miRNAs (miR-140-5p, miR-152-3p, miR-30e-5p, miR-324-3p, miR-335-3p, miR-19a-3p, miR-19b-3p, and miR-550a-3p) had AUC values of 0.9 for the classification of fracture patients, correctly discriminating between fractured patients and healthy subjects [64].

Some of these miRNAs have been already involved in the regulation of bone metabolism. In vitro, miR-140-5p directly represses the expression of BMP2, inhibiting the differentiation of human mesenchymal cell lines towards the osteoblastic linage [65]. miR-19a and miR-19b are both part of a cluster called miR-17-92, whose haploinsufficiency in mice causes impaired ALP activity and impaired bone calcification [66]. Furthermore, a significant and peculiar regulation in the serum of osteoporotic patients with recent fractures (compared to healthy subjects) was detected concerning miR-10b-5p, miR-133b, miR-22-3p, and let-7 g-5p, but only the last 2 were validated [67].

Indeed, both miR-22 and let-7g-5p were previously demonstrated to either promote or inhibit osteogenic differentiation. miR-22 was found to regulate the fate of MSCs, promoting the differentiation towards osteoblasts and inhibiting adipogenesis in human adipose tissue-derived mesenchymal stem cells (hADMSCs) through the downregulation of HDAC6, a repressor of Runx2, thus underlying an important role in the balance of adipogenesis and osteogenesis [68]. In vitro, let-7 significantly promoted osteogenesis and counteracted adipogenesis of MSCs, by targeting high-mobility group AT-hook 2 (HMGA2) expression [69].

More recently, Yavropoulou et al. described other miRNAs also known to modulate bone turnover (e.g., miR-23a, miR-29a-3p, miR-124-3p, and miR-2861) that were significantly deregulated in the serum of patients with low bone mass and vertebral fractures compared with controls [62]. For example, miR-23a significantly inhibits TNF-*α*-induced apoptosis in MC3T3-E1 cells (osteoblastic line) by targeting Fas, a proapoptotic protein, and promotes osteogenic differentiation by targeting Runx2 expression in ATDC5 cells [70, 71]. Similarly, miR-29a regulates HDAC4 expression, displaying protective effects from glucocorticoid-induced bone loss by modulating *β*-catenin accumulation and OB differentiation [72]. In BMSCs, miR-124 inhibits the differentiation toward the osteogenic lineage, favoring adipogenic differentiation, thereby suppressing in vivo bone formation by binding to Dlx5, Dlx3, and Dlx2 [73]. Moreover, miR-124 is a negative regulator of osteoclastogenesis in mouse BMMs. It reduces the expression of NFATc1 protein induced by RANKL stimulation and inhibits the OC precursors by targeting expression of RhoA and Rac1, GTPases involved in cellular motility [74].

Interestingly, a previous study also identified miR-2861 as a promoter of osteoblast differentiation by targeting histone deacetylase 5, resulting in increased Runx2 protein levels in ST2 cells induced with BMP-2 [75]. In this study, two related osteoporotic patients presented a mutation on miR-2861 that impaired its expression, suggesting that the downregulation of this miRNA may contribute to the disease, although, Yavropoulou et al. [62] found it upregulated in osteoporotic patients.

### 4.2. Combination of miRNAs Identifies Osteoporotic Patients with Fractures

Trying to identify single miRNAs as significant biomarkers for osteoporosis, or any other disease, could be laborious, difficult to apply to various populations, and may not be sufficient to discriminate complex aspects of the disease. As suggested by a recent analysis by Heilmeier et al., a more specific predictive value on fracture risk could be achieved through combinations of different miRNAs [76]. Stronger biomarkers could statistically be identified clustering miRNAs that show differential expression in osteoporosis; a multigene approach could be applied to identify clusters of miRNAs with higher discriminating values [77]. Regarding osteoporosis, few studies have investigated this aspect, but this approach has already been applied to many other disease, including cancer, for example, the miR-183/182/96 cluster expression correlated with metastasis and poor clinical outcome in breast cancer patients [78].

In the study of Heilmeier et al., which also included subjects with type 2 diabetes and fractures, 23 differentially expressed miRNAs were found in osteoporotic fractured patients and a specific signature of 4 miRNAs, composed of miR-382-3p, miR-188-3p, miR-942, and miR-330-3p, was able to correctly discriminate between postmenopausal women with osteoporotic fractures and postmenopausal women without fractures with the highest AUC value (0.991). Interestingly, a partially different signature was identified in subjects affected by type 2 diabetes and fractures. In fact, only miR-382-3p was downregulated both in diabetic and non diabetic patients with fractures, while 3 additional miRNAs (miRNA-96-5p, miRNA-181-5p and miRNA-550a-5p, all upregulated) were specific for the signature linked to fractures in diabetes. From the experimental point of view, in vitro functional studies demonstrated that in human adipose tissue-derived mesenchymal stem cells, miR-382 is able to significantly enhance osteogenic differentiation [76].

Another cluster worth mentioning is cluster miR-23, composed of miR-23a, miR-27a, miR-24a. Several studies, in vitro and in vivo, have uncovered important functions of this cluster in bone cells, but to date, no clinical studies investigated its potential role as a biomarker for osteoporosis [79]. Given that the single component of miR-23 was indeed found differentially expressed in osteoporotic patients, as described in the previous paragraphs, it would be interesting to explore its potential as a combination.

## 5. Conclusions and Future Perspectives

It is well established that in the next future miRNAs could become valid biomarkers for several diseases, for diagnostic purposes, prediction of complications, and response to treatment. However, in bone pathologies and more specifically in osteoporosis, we are only at the beginning in this field. To date, the available evidences are indeed promising and have been able to uncover interesting miRNAs that are not only involved in specific functions and roles in the bone biology (as demonstrated in experimental observations), but are also potentially capable of discriminating osteoporotic patients from controls, thus conferring them a relevant value for both medicine and basic research. Of course, we are still far from identifying a strong biomarker for osteoporosis that could be applied to all cohorts of patients, especially because of to the low number of studies on circulating miRNAs and their methodological discrepancies, as mentioned before. In the future, given the potential of such biomarkers, it will be extremely important to perform a further validation of these and other miRNAs in prospective studies of larger population-based samples, taking also into account relevant variables that could influence miRNA expression profile, such as diet or age.

## Figures and Tables

**Table 1:** Differentially expressed miRNAs in osteoporotic patients that are commonly or uncommonly expressed in serum and plasma (miRBase V18.0 nomenclature).

Common miRNAs	Uncommon miRNAs
Let-7g	miR-100
miR-122	miR-10b-5p
miR-125	miR-124a
miR-133a	miR-130b
miR-140-5p	miR-151a-3p
miR-142-3p	miR-151b
miR-148a	miR-181
miR-152	miR-188-3p
miR-21	miR-194-5p
miR-22	miR-19a
miR-23a	miR-19b
miR-24	miR-2861
miR-27a	miR-31
miR-29a	miR-330-3p
miR-30b	miR-382
miR-30e	miR-550a-3p
miR-324-3p	miR-590-5p
miR-335	miR-660-5p
miR-423-5p	miR-942
miR-93	miR-96

**Table 2:** miRNA dysregulated in serum of osteoporotic patients and their function.

miRNA	Expression in OP patients	Biological function	Target	Ref.
Let-7g-5p	Downregulated	Promotes osteogenesis and ectopic bone formation and suppresses adipogenesis	HMGA2	[69]

miR-10b-5p	Upregulated	Potent inhibitor of OB differentiation	Bcl6	[80]

miR-19a miR-19b	Downregulated	Part of a cluster of miRNAs that probably maintains OB in undcifferentiated state	Undetected	[64]

miR-21	Upregulated	Promotes OB differentiation and mineralization	Smad7	[43]
		Essential for OC formation and OC bone-resorbing activity, preserves OCs from apoptosis	FasL, PDCD4	[41, 42]

miR-22-3p	Downregulated	Important regulator of the balance between adipogenic and osteogenic differentiation. Promoter of OB differentiation	HDAC6	[68]

miR-23a	Upregulated	Potent inhibitor of OB apoptosis and promoter of osteogenic differentiation	Fas, Runx2	[70, 71]

miR-24	Upregulated	Inhibits OB differentiation and mineralization	Tcf-1	[81]

miR-27	Downregulated	Promotes OB differentiation by inhibition of the suppressor of *β*-catenin	APC	[56]

miR-29a-3p	Downregulated	Promotes OB differentiation by repressing endogenous levels of Wnt signaling antagonists	DKK1, Kremen2, sFRP2	[82]

miR-30 family	Downregulated	Downregulated during OB differentiation are considered negative regulators of osteogenesis. One component of this family promotes adipogenesis rather than OB genesis	Smad1, Runx2, LRP6	[59]

miR-100	Upregulated	Negative regulator of OB differentiation	BMPR2	[83]

miR-124	Upregulated	Enhances the adipogenic differentiation of BMSCs and inhibits osteogenesis. It is a suppressor of bone formation	Dlx5, Dlx3, Dlx2	[73]
		Negative regulator of RANKL-dependent OC genesis. It inhibits the migration of OC precursors	NFATc1, RhoA, Rac1	[73]

miR-125	Upregulated	Negative regulator of OB precursor proliferation and OB differentiation in the early stages	ErbB2	[63]

miR-140-5p	Downregulated	Downregulated during osteogenesis. It is a suppressor of OB differentiation	BMP2	[65]

miR-142	Downregulated	Positively regulates OB precursor differentiation by activating Wnt signaling	APC	[60]

miR-148a	Upregulated	Negative regulator of adipogenesis and promoter of OB differentiation	Kdm6b	[46]
		Promotes OC genesis and increased the bone resorption area on dentin slices	MAFB	[45]

miR-2861	Upregulated	Promoter of OB differentiation	HDAC5	[75]

**Table 3:** miRNA dysregulated in plasma of osteoporotic patients and their function.

miRNA	Expression	Function	Target	Reference
miR-21	Downregulated	miR-21 promotes OB differentiation and mineralization	Smad7	[43]
miR-21		Essential for OC formation and OC bone-resorbing activity, preserves OCs from apoptosis	FasL, PDCD4	[42]

miR-31	Upregulated	Promotes OC formation and activity by promoting the formation of the peripheral acting ring	RhoA	[54]

miR-133a	Upregulated	Downregulated during OB differentiation, it is a suppressor of osteogenesis	Runx2	[57]

miR-148a	Upregulated	Promotes OC genesis and increased the bone resorption area on dentin slices	MAFB	[45]

## References

[1] Rachner T. D., Khosla S., Hofbauer L. C. (2011). Osteoporosis: now and the future.

[2] Gennari L., Rotatori S., Bianciardi S., Nuti R., Merlotti D. (2016). Treatment needs and current options for postmenopausal osteoporosis.

[3] Gennari L., Rotatori S., Bianciardi S., Gonnelli S., Nuti R., Merlotti D. (2015). Appropriate models for novel osteoporosis drug discovery and future perspectives.

[4] Seeman E., Delmas P. D. (2006). Bone quality--the material and structural basis of bone strength and fragility.

[5] Martin T. J., Seeman E. (2008). Bone remodelling: its local regulation and the emergence of bone fragility.

[6] Raisz L. G. (2005). Pathogenesis of osteoporosis: concepts, conflicts and prospects.

[7] Sambrook P., Cooper C. (2006). Osteoporosis.

[8] Cummings S. R., Browner W., Black D. M. (1993). Bone density at various sites for prediction of hip fractures.

[9] Sozen T., Ozisik L., Basaran N. C. (2017). An overview and management of osteoporosis.

[10] Garnero P. (2014). New developments in biological markers of bone metabolism in osteoporosis.

[11] Hackl M., Heilmeier U., Weilner S., Grillari J. (2016). Circulating microRNAs as novel biomarkers for bone diseases - complex signatures for multifactorial diseases?.

[12] Unnanuntana A., Gladnick B. P., Donnelly E., Lane J. M. (2010). The assessment of fracture risk.

[13] Vestergaard P., Rejnmark L., Mosekilde L. (2005). Osteoporosis is markedly underdiagnosed: a nationwide study from Denmark.

[14] Gennari L., Bianciardi S., Merlotti D. (2017). MicroRNAs in bone diseases.

[15] Roberts S. B., Wootton E., De Ferrari L., Albagha O. M., Salter D. M. (2015). Epigenetics of osteoarticular diseases: recent developments.

[16] Jacquier A. (2009). The complex eukaryotic transcriptome: unexpected pervasive transcription and novel small RNAs.

[17] Tay Y., Rinn J., Pandolfi P. P. (2014). The multilayered complexity of ceRNA crosstalk and competition.

[18] Esquela-Kerscher A., Slack F. J. (2006). Oncomirs - microRNAs with a role in cancer.

[19] Tang G., Reinhart B. J., Bartel D. P., Zamore P. D. (2003). A biochemical framework for RNA silencing in plants.

[20] Palatnik J. F., Allen E., Wu X. (2003). Control of leaf morphogenesis by microRNAs.

[21] Llave C., Xie Z., Kasschau K. D., Carrington J. C. (2002). Cleavage of *Scarecrow-like* mRNA targets directed by a class of *Arabidopsis* miRNA.

[22] Yekta S., Shih I. H., Bartel D. P. (2004). MicroRNA-directed cleavage of *HOXB8* mRNA.

[23] Hutvàgner G., Zamore P. D. (2002). A microRNA in a multiple-turnover RNAi enzyme complex.

[24] Pillai R. S., Bhattacharyya S. N., Artus C. G. (2005). Inhibition of translational initiation by Let-7 microRNA in human cells.

[25] Tong X., Gu P. C., Xu S. Z., Lin X. J. (2015). Long non-coding RNA-DANCR in human circulating monocytes: a potential biomarker associated with postmenopausal osteoporosis.

[26] Jia Q., Jiang W., Ni L. (2015). Down-regulated non-coding RNA (lncRNA-ANCR) promotes osteogenic differentiation of periodontal ligament stem cells.

[27] Yavropoulou M. P., Yovos J. G. (2018). The ‘dark matter’ of DNA and the regulation of bone metabolism: the role of non-coding RNAs.

[28] Zhu L., Xu C. P. C. (2013). Downregulated LncRNA-ANCR promotes osteoblast differentiation by targeting EZH2 and regulating Runx2 expression.

[29] Liang W. C., Fu W. M., Wang Y. B. (2016). H19 activates Wnt signaling and promotes osteoblast differentiation by functioning as a competing endogenous RNA.

[30] Wang Q., Li Y., Zhang Y. (2017). LncRNA MEG3 inhibited osteogenic differentiation of bone marrow mesenchymal stem cells from postmenopausal osteoporosis by targeting miR-133a-3p.

[31] Kobayashi T., Lu J., Cobb B. S. (2008). Dicer-dependent pathways regulate chondrocyte proliferation and differentiation.

[32] Gaur T., Hussain S., Mudhasani R. (2010). Dicer inactivation in osteoprogenitor cells compromises fetal survival and bone formation, while excision in differentiated osteoblasts increases bone mass in the adult mouse.

[33] Wang K., Yuan Y., Cho J. H., McClarty S., Baxter D., Galas D. J. (2012). Comparing the micro-RNA spectrum between serum and plasma.

[34] Blondal T., Jensby Nielsen S., Baker A. (2013). Assessing sample and miRNA profile quality in serum and plasma or other biofluids.

[35] Wahid F., Khan T., Kim Y. Y. (2014). MicroRNA and diseases: therapeutic potential as new generation of drugs.

[36] Li Y., Kowdley K. V. (2012). MicroRNAs in common human diseases.

[37] Wang J., Chen J., Sen S. (2016). MicroRNA as biomarkers and diagnostics.

[38] Sebastiani G., Nigi L., Grieco G. E., Mancarella F., Ventriglia G., Dotta F. (2017). Circulating microRNAs and diabetes mellitus: a novel tool for disease prediction, diagnosis, and staging?.

[39] Wang J., Zhang K. Y., Liu S. M., Sen S. (2014). Tumor-associated circulating microRNAs as biomarkers of cancer.

[40] Seeliger C., Karpinski K., Haug A. T. (2014). Five freely circulating miRNAs and bone tissue miRNAs are associated with osteoporotic fractures.

[41] Sugatani T., Vacher J., Hruska K. A. (2011). A microRNA expression signature of osteoclastogenesis.

[42] Sugatani T., Hruska K. A. (2013). Down-regulation of miR-21 biogenesis by estrogen action contributes to osteoclastic apoptosis.

[43] Li H., Yang F., Wang Z., Fu Q., Liang A. (2015). MicroRNA-21 promotes osteogenic differentiation by targeting small mothers against decapentaplegic 7.

[44] Yano M., Inoue Y., Tobimatsu T. (2012). Smad7 inhibits differentiation and mineralization of mouse osteoblastic cells.

[45] Cheng P., Chen C., He H. B. (2013). miR-148a regulates osteoclastogenesis by targeting V-maf musculoaponeurotic fibrosarcoma oncogene homolog B.

[46] Tian L., Zheng F., Li Z. (2017). miR-148a-3p regulates adipocyte and osteoblast differentiation by targeting lysine-specific demethylase 6b.

[47] Li H., Wang Z., Fu Q., Zhang J. (2014). Plasma miRNA levels correlate with sensitivity to bone mineral density in postmenopausal osteoporosis patients.

[48] Bedene A., Mencej Bedrač S., Ješe L. (2016). MiR-148a the epigenetic regulator of bone homeostasis is increased in plasma of osteoporotic postmenopausal women.

[49] Ma W., Zhang X., Chai J., Chen P., Ren P., Gong M. (2014). Circulating miR-148a is a significant diagnostic and prognostic biomarker for patients with osteosarcoma.

[50] Meng J., Zhang D., Pan N. (2015). Identification of miR-194-5p as a potential biomarker for postmenopausal osteoporosis.

[51] Weilner S., Schraml E., Wieser M. (2016). Secreted microvescicular miR-31 inhibits osteogenic differentiation of mesenchymal stem cells.

[52] Li J., He X., Wei W., Zhou X. (2015). MicroRNA-194 promotes osteoblast differentiation via down regulating STAT1.

[53] Jeong B. C., Kang I. H., Hwang Y. C., Kim S. H., Koh J. T. (2014). MicroRNA-194 reciprocally stimulates osteogenesis and inhibits adipogenesis via regulating COUP-TFII expression.

[54] Mizoguchi F., Murakami Y., Saito T., Miyasaka N., Kohsaka H. (2013). miR-31 controls osteoclast formation and bone resorption by targeting RhoA.

[55] You L., Pan L., Chen L., Gu W., Chen J. (2016). MiR-27a is essential for the shift from osteogenic differentiation to adipogenic differentiation of mesenchymal stem cells in postmenopausal osteoporosis.

[56] Wang T., Xu Z. (2010). miR-27 promotes osteoblast differentiation by modulating Wnt signaling.

[57] Li Z., Hassan M. Q., Volinia S. (2008). A microRNA signature for a BMP2-induced osteoblast lineage commitment program.

[58] Chen J., Li K., Pang Q. (2016). Identification of suitable reference gene and biomarkers of serum miRNAs for osteoporosis.

[59] Wu T., Zhou H., Hong Y., Li J., Jiang X., Huang H. (2012). miR-30 family members negatively regulate osteoblast differentiation.

[60] Hu W., Ye Y., Zhang W., Wang J., Chen A., Guo F. (2013). miR-142-3p promotes osteoblast differentiation by modulating Wnt signaling.

[61] Panach L., Mifsut D., Tarin J. J., Cano A., Garcia-Perez M. A. (2015). Serum circulating microRNAs as biomarkers of osteoporotic fracture.

[62] Yavropoulou M. P., Anastasilakis A. D., Makras P., Tsalikakis D. G., Grammatiki M., Yovos J. G. (20172016). Expression of microRNAs that regulate bone turnover in the serum of postmenopausal women with low bone mass and vertebral fractures.

[63] Mizuno Y., Yagi K., Tokuzawa Y. (2008). miR-125b inhibits osteoblastic differentiation by down-regulation of cell proliferation.

[64] Kocijan R., Muschitz C., Geiger E. (2016). Circulating microRNA signatures in patients with idiopathic and postmenopausal osteoporosis and fragility fractures.

[65] Hwang S., Park S. K., Lee H. Y. (2014). miR-140-5p suppresses BMP2-mediated osteogenesis in undifferentiated human mesenchymal stem cells.

[66] Zhou M., Ma J., Chen S., Chen X., Yu X. (2014). MicroRNA-17-92 cluster regulates osteoblast proliferation and differentiation.

[67] Weilner S., Skalicky S., Salzer B. (2015). Differentially circulating miRNAs after recent osteoporotic fractures can influence osteogenic differentiation.

[68] Huang S., Wang S., Bian C. (2012). Upregulation of miR-22 promotes osteogenic differentiation and inhibits adipogenic differentiation of human adipose tissue-derived mesenchymal stem cells by repressing *HDAC6* protein expression.

[69] Wei J., Li H., Wang S. (2014). *Let-7* enhances osteogenesis and bone formation while repressing adipogenesis of human stromal/mesenchymal stem cells by regulating HMGA2.

[70] Dong J., Cui X., Jiang Z., Sun J. (2013). MicroRNA-23a modulates tumor necrosis factor-alpha-induced osteoblasts apoptosis by directly targeting Fas.

[71] Zhang Y., Xie R. L., Croce C. M. (2011). A program of microRNAs controls osteogenic lineage progression by targeting transcription factor Runx2.

[72] Ko J. Y., Chuang P. C., Chen M. W. (2013). MicroRNA-29a ameliorates glucocorticoid-induced suppression of osteoblast differentiation by regulating *beta*-catenin acetylation.

[73] Qadir A. S., Um S., Lee H. (2015). miR-124 negatively regulates osteogenic differentiation and in vivo bone formation of mesenchymal stem cells.

[74] Lee Y., Kim H. J., Park C. K. (2013). MicroRNA-124 regulates osteoclast differentiation.

[75] Li H., Xie H., Liu W. (2009). A novel microRNA targeting HDAC5 regulates osteoblast differentiation in mice and contributes to primary osteoporosis in humans.

[76] Heilmeier U., Hackl M., Skalicky S. (2016). Serum miRNA signatures are indicative of skeletal fractures in postmenopausal women with and without type 2 diabetes and influence osteogenic and adipogenic differentiation of adipose tissue-derived mesenchymal stem cells in vitro.

[77] Yang Y., Huang N., Hao L., Kong W. (2017). A clustering-based approach for efficient identification of microRNA combinatorial biomarkers.

[78] Song C., Zhang L., Wang J. (2016). High expression of microRNA-183/182/96 cluster as a prognostic biomarker for breast cancer.

[79] Zeng H. C., Bae Y., Dawson B. C. (2017). MicroRNA miR-23a cluster promotes osteocyte differentiation by regulating TGF-*β* signalling in osteoblasts.

[80] Yang J., Wang S., Wang F. (2017). Downregulation of miR-10b promotes osteoblast differentiation through targeting Bcl6.

[81] Zhao W., Wu C., Dong Y., Ma Y., Jin Y., Ji Y. (2015). MicroRNA-24 regulates osteogenic differentiation via targeting T-cell factor-1.

[82] Franceschetti T., Kessler C. B., Lee S. K., Delany A. M. (2013). miR-29 promotes murine osteoclastogenesis by regulating osteoclast commitment and migration.

[83] Zeng Y., Qu X., Li H. (2012). MicroRNA-100 regulates osteogenic differentiation of human adipose-derived mesenchymal stem cells by targeting BMPR2.

